# Mobile-bearing versus fixed-bearing total knee arthroplasty: a meta-analysis of randomized controlled trials.

**DOI:** 10.1007/s00590-021-02999-x

**Published:** 2021-05-22

**Authors:** Ashraf T. Hantouly, Abdulaziz F. Ahmed, Osama Alzobi, Ammar Toubasi, Motasem Salameh, Aissam Elmhiregh, Shamsi Hameed, Ghalib O. Ahmed, Abtin Alvand, Mohammed Al Ateeq Al Dosari

**Affiliations:** 1 Department of Orthopaedic Surgery, Surgical Specialty Center, Hamad Medical Corporation, Doha, Qatar; 2 Nuffield Department of Orthopaedics, Rheumatology & Musculoskeletal Sciences, University of Oxford, Oxford, UK

**Keywords:** Mobile, Fixed, Bearing, Total knee, Arthroplasty, Meta-analysis, Systematic review

## Abstract

**Objective:**

The purpose of this study was to perform a meta-analysis comparing mobile-bearing with fixed-bearing total knee arthroplasty (TKA) in terms of all-cause revision rates, aspetic loosening, knee functional scores, range of motion and radiographic lucent lines and osteolysis.

**Methods:**

PubMed, Cochrane Library, Google Scholar and Web of Science were searched up to January 2020. Randomized controlled trials that compared primary mobile-bearing with fixed-bearing TKA, reporting at least one of the outcomes of interest, at a minimum follow-up of 12 months were included. All outcomes of interest were pooled at short-term (< 5 years), mid-term (5 to 9 years) and long-term (> = 10 years) follow-up intervals.

**Results:**

A total of 70 eligible articles were included in the qualitative and statistical analyses. There was no difference between mobile-bearing or fixed-bearing TKA at short-term, mid-term and long-term follow-ups in all outcome measures including all-cause revision rate, aseptic loosening, oxford knee score, knee society score, Hospital for Special Surgery score, maximum knee flexion, radiographic lucent lines and radiographic osteolysis.

**Conclusion:**

The current level of evidence demonstrated that both mobile-bearing and fixed-bearing designs achieved excellent outcomes, yet it does not prove the theoretical advantages of the mobile-bearing insert over its fixed-bearing counterpart. The use of either design could therefore be supported based on the outcomes assessed in this study.

Level of Evidence: Level II, Therapeutic

**Supplementary information:**

The online version contains supplementary material available at (10.1007/s00590-021-02999-x).

## Introduction

The design of the polyethylene insert has been debated numerously in the literature [22]. Fixed-bearing designs, which provide rigid fixation of the polyethylene insert within the tibial implant, have demonstrated satisfactory outcomes and long-term survival rates [1, 38, 45, 64]. However, implant loosening in fixed-bearing designs was theoretically attributed to higher contact stresses and polyethylene wear rates [20, 75], which motivated the pursuit of improved TKA designs. Mobile-bearing polyethylene designs were developed to mitigate the drawbacks of fixed-bearing TKA through improving the conformity, lowering contact stresses with the aim of mimicking the kinematics of the native knee [16]. However, these advantages are theoretical and yet to be fully proven in vivo. Furthermore, mobile-bearing TKA can introduce unique complications such as bearing dislocation [5].

Earlier meta-analyses have reported superior results with the mobile-bearing TKA [13, 85]. Subsequent meta-analysis with mid-term follow-up had refuted such findings without any significant difference between mobile-bearing and fixed-bearing TKA [55, 81]. However, in June 2020 two recent meta-analyses with a limited number of studies presented further contradicting results, with one meta-analysis supporting long-term clinical outcomes in favor of mobile bearing, whereas the other meta-analysis refuted such findings [15, 84]. Therefore, controversy continues to exist regarding the superiority of mobile-bearing over fixed-bearing designs. This study aimed to provide an updated meta-analysis comparing mobile-bearing versus fixed-bearing TKA using a multi-modal method of outcomes to include overall revision rates, aseptic loosening, clinical as well as radiological outcomes. Our hypothesis was that no significant differences exist in all outcomes between the mobile-bearing and the fixed-bearing designs.

## Materials and methods

This meta-analysis was conducted with adherence to the Preferred Reporting Items for Systematic Reviews and Meta-Analyses (PRISMA) [54]. The focus was randomized controlled trials that compared mobile-bearing with fixed-bearing TKA. The primary outcome was the all-cause revision rate. The secondary outcomes were aseptic loosening rates, knee functional scores, maximum knee flexion, radiographic lucent lines and osteolysis.

### Eligibility criteria

The inclusion criteria were randomized controlled trials that compared primary mobile-bearing with fixed-bearing TKA, reporting at least one of the outcomes of interest, a minimum follow-up of 12 months. Exclusion criteria were inaccessible full-text, abstracts and studies reporting outcomes of interest but with unextractable data for meta-analytic comparisons. Articles published in English were only sought. Studies that reported the same sample population were not excluded if the follow-up intervals were different. The exclusion criteria were non-randomized clinical trials and studies with a population reported in a previous study with an overlapping follow-up interval.

### Information sources and search strategy

PubMed, Cochrane Library, Google Scholar and Web of Science were searched till January 2020.

The search strategy involved the use of the following keywords that involved synonyms of “total knee arthroplasty” AND “mobile bearing” AND “fixed bearing” AND “randomized controlled trials.”

Studies were screened by titles and abstracts. A full-text review was performed if a study matched the eligibility criteria. Furthermore, the references of each eligible article were manually searched to ensure eligible studies were not missed. The search strategy was performed by three authors independently. Any disagreement between the three authors in the search strategy was resolved by the senior author.

### Data collection process and data items

The data items that were collected included: the first author’s surname, study year, study location, age, sex, number of patients, type of prosthetic bearing used (mobile-bearing or fixed-bearing), the specific type of mobile-bearing prosthesis (rotating platform, rotating platform and gliding, and meniscal bearing), patella resurfacing, follow-up timepoints, all-cause revision rates, Oxford Knee Scores (OKS), Knee Society Scores (KSS), the Hospital for Special Surgery (HSS) knee scores, reported maximum knee flexion, radiographic radiolucent lines, radiographic osteolysis and rates of aseptic loosening. The OKS was transformed into the 0–48 scale to facilitate data synthesis. The Western Ontario and McMaster Universities Arthritis Index was not collected as it was reported variably among studies with the 0–96 Likert scores or the 0–100 visual analog scales. Data collection forms were used independently by three authors, with any arising disagreement in the collected data being resolved by the senior author.

### Risk of bias in individual studies

The qualitative analysis was performed with the revised Cochrane risk-of-bias tool for randomized trials (RoB 2) [74]. The tool contains five domains that assesses the randomization, adherence to intended treatments, missing outcomes, measurement bias and reporting bias. Each study was assessed with the RoB 2 by three authors independently, and the final rating of each study was reviewed by the three authors and the senior author to arrive at a consensus.

### Statistical analysis

Analysis was performed with the use of Stata/IC (StataCorp. 2019. Stata Statistical Software: Release 16. College Station, TX: StataCorp LLC.). The outcomes were estimated with the use of 95% confidence interval (CI). The risk ratio (RR) was utilized for dichotomous outcomes such as the revision rates and the aseptic loosening rates. The mean difference (MD) was used for expressing continuous outcomes such as the OKS, the KSS and the HSS knee score. The Hedge’s G mean difference was used for maximum knee flexion due to potential variability in the range of motion measurements. The outcome measures of interest were pooled at three different follow-up intervals at short term (<5 years), mid-term (5 to 9 years) and long term (>=10 years). The meta-analytic models were based on random effects (RE) with the use of the DerSimonian-Laird method as a heterogeneity variance estimator [17]. The formulas developed by Hozo et al.[31] were used in studies that reported medians instead of means and ranges instead of standard deviations (SD).

## Results

### Study selection

**Fig. 1:**
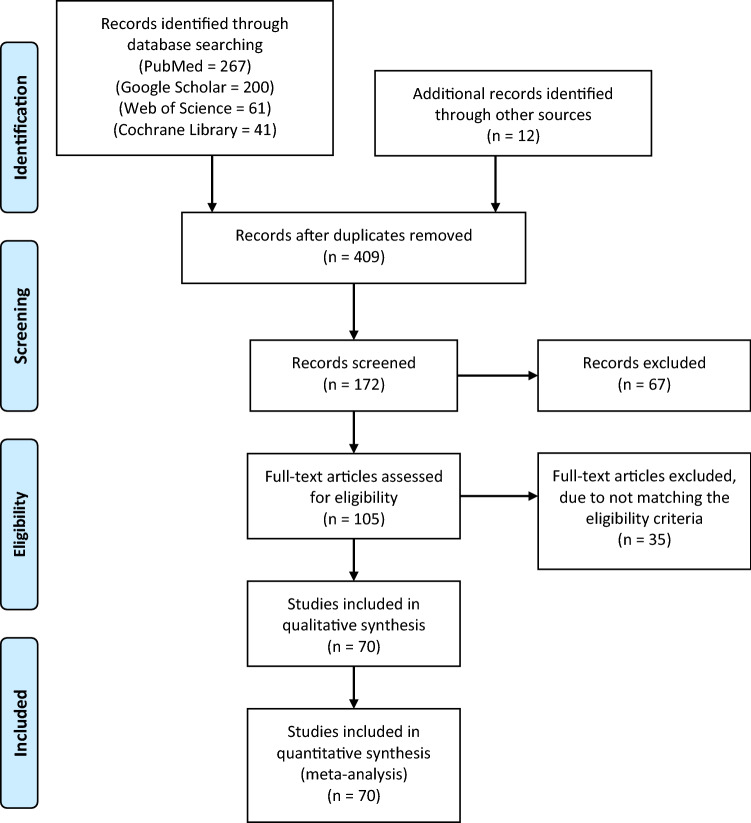
Search strategy flowchart

The search strategy resulted in 581 (569 articles from database search and 12 articles from manual references search) articles, of which 409 articles were excluded due to duplications. Subsequently, a total of 172 articles were screened by titles and abstracts, of which 67 articles were excluded. This resulted in a total of 105 articles that were eligible for full-text reviews, of which 35 articles were excluded. Thus, a total of 70 articles were included in the qualitative and statistical analyses. The PRISMA flowchart is displayed in Fig. 1.

### Study characteristics

Among the 70 included studies, 4968 patients underwent mobile-bearing TKA and 5034 patients underwent fixed-bearing TKA. The most utilized TKA implant was PFC Sigma® (DePuy) in 34.3% of all studies. A posterior-stabilized (PS) implant was routinely used in 60% of studies, whereas a cruciate-retaining (CR) design was routinely used in 25.7%. The rest of the studies used either CR or PS designs (4.3%) depending on the total knee system utilized, and 10% of studies did not specify whether the posterior cruciate ligament was sacrificed. The mobile-bearing designs used were a rotating platform in 81.4%, rotating and anterior–posterior gliding in 11.4% and meniscal bearing in 2.86%. Patella resurfacing was performed routinely in 48.57% of studies, unresurfaced in 22.86% and selectively resurfaced on a case-by-case basis in 17.14%. Study characteristics are summarized in Table 1.

**Table 1:** Characteristics of included studies

Study	Country	LoE	Group	Knees (N)	Age	Females (%)	TKA design	MB type	Cruciate design	Patella resurfacing	Follow-up
Killen, 2019[36]	USA	Level I	MB	30	76.57	66.6%	PFC Sigma; DePuy	RP	CR or PS	All resurfaced	13.95
			FB	21	76.79						
Tiwari 2019[78]	South Korea	Level I	MB	260	69.7	94.6%	E.Motion PS-Pro; B.Braun-Aesculap	RP	PS	All resurfaced	2
			FB	133			Genesis II; Smith & Nephew				
Sappey-Marinier 2019[70]	France	Level I	MB	65	71	58.7%	HLS Noetos knee prosthesis; Tornier	RP	PS	All resurfaced	7.4
			FB	64							
Park 2019[62]	South Korea	Level II	MB	70	69.5	94.2%	ACS; Implantcast	-	PS	All resurfaced	4
			FB	70	68.9						
Kim 2019[39]	South Korea	Level I	MB	164	63	86.5%	NexGen LPS-Flex; Zimmer	RP	PS	All resurfaced	17
			FB	164							
Kim 2018[43]	South Korea	Level I	MB	92	61.5	81.5%	NexGen LPS-Flex; Zimmer	RP	PS	All resurfaced	12
			FB	92							
Van Hamersveld 2018[82]	Netherlands	Level II	MB	23	67.5	76.1%	Triathalon; Stryker	RP	PS	None	6
			FB	23	68						
Powell 2018[64]	New Zealand	Level I	MB	91	65.5	43.7%	PFC Sigma; DePuy	RP	CR	At surgeon's discretion	14
			FB	99							
Chaudhry 2018 [14]	India	Level II	MB	50	58.7	54.5%	PFC Sigma; DePuy	RP	PS	At surgeon's discretion	6–8
			FB	60	57.6						
Abdel 2018[1]	USA	Level I	MB	55	67.4	65.6%	PFC Sigma; DePuy	RP	PS	All resurfaced	10
			FB	114	67						
Amaro 2017[3]	Brazil	Level I	MB	32	65.2	71.9%	NR	RP	PS	None	2
			FB	32	66.2						
Feczko 2017[18]	Netherlands	Level II	MB	42	NR	NR	Scorpio; Stryker	RP	PS	All resurfaced	5
			FB	48							
Schotanus 2017[71]	Netherlands	Level I	MB	20	61.9	41.4%	Vanguard; Zimmer Biomet	RP	NR	NR	3
			FB	21	67.1						
Baktir 2016[7]	Turkey	Level I	MB	47	64.9	88.2%	TC-PLUS; Smith & Nephew	RP	CR	None	8
			FB	46	64.7		Maxim; Biomet				
Artz 2015[4]	UK	Level II	MB	104	61.7	51%	Rotaglide; Corin	RP + AP gliding	NR	NR	2
			FB	102	61.6						
Minoda 2015[53]	Japan	Level I	MB	46	74.3	88.3%	Vanguard; Zimmer Biomet	RP	PS	NR	2
			FB	48	75.7						
Van De Groes 2015[80]	Netherlands	Level II	MB	24	66.5	49%	PFC Sigma; DePuy	RP	PS	None	1.2
			FB	23	66.2				CR		1.6
Fransen 2015[21]	Netherlands	Level I	MB	114	65.7	69.6%	Genesis II; Smith & Nephew	RP or RT/AP gliding	CR	At surgeons discretion	5
			FB	123	65.8						
Tjørnild 2015[79]	Denmark	Level II	MB	27	66	54%	PFC Sigma; DePuy	RP	CR	All resurfaced	2
			FB	28							
Marques 2015[51]	Germany	Level I	MB	48	69.4	73%	Columbus, BBraun Aesculap	RP	CR	None	4
			FB	52	68.9						
Bailey 2015[6]	UK	Level I	MB	161	69.2	57.1%	PFC Sigma; DePuy	RP	CR	At surgeons discretion	2
			FB	170	70.1						
Okamoto 2014[60]	Japan	Level I	MB	20	76	85%	NexGen LPS-Flex; Zimmer	RP	PS	None	1
			FB	20	78						
Breugem 2014[12]	Netherlands	Level II	MB	29	78	65.2%	NexGen Legacy; Zimmer	RP	PS	All resurfaced	7.9
			FB	40	80						
Ferguson 2014[19]	UK	Level II	MB	176	70.2	53.1%	PFC Sigma; DePuy	RP	PS	At surgeons discretion	2
			FB	176	69.8						
Breeman 2013[10]	UK	Level II	MB	276	69	60.1%	Non-specific	Non-specific		At surgeons discretion	5
			FB	263							
Nieuwenhuijse 2013[58]	Netherlands	Level I	MB	37	66.8–68.7	80.8%	NexGen LPS-Flex/LPS; Zimmer	RP	PS	At Surgeons discretion	5
			FB	41	68.5–72.2						
Prasad 2013[65]	India	Level II	MB	16	63.75	62.5%	Exactech; Optetrek	RP	PS	None	1
			FB	16	63.68				CR		
Radetzki 2013[67]	Germany	Level II	MB	17	66..5	53.8%	NexGen LPS-Flex; Zimmer	RP	PS	All resurfaced	10.8
			FB	22	65.6		NexGen LPS; Zimmer				
Kim 2012 [38]	South Korea	Level I	MB	108	45	76.9%	LCS; DePuy	RP	PS	All resurfaced	16.8
			FB	108			AMK; DePuy		CR		
Scuderi 2012[72]	USA & Canada	Level I	MB	152	63.7	58.4%	NexGen LPS-Flex; Zimmer	RP	PS	All resurfaced	4
			FB	141	63.4						
Pijls 2012[63]	Netherlands	Level II	MB	21	64	81%	Interax; Stryker	RP + AP gliding	PS	NR	10–12
			FB	21	66						
Nutton 2012[59]	UK	Level I	MB	36	68.3	51.3%	PFC Sigma; DePuy	RP	PS	None	1
			FB	40	69.8				CR		
Mahoney 2012[50]	USA	Level II	MB	252	66	63.9%	Scorpio – Stryker	RP	PS	All resurfaced	2
			FB	255							
Jolles 2012[34]	Switzerland	Level I	MB	26	67.1	58%	NexGen LPS-Flex; Zimmer	RP	PS	All resurfaced	2
			FB	29	70.2						
Lizaur-Utrilla 2012[49]	Spain	Level I	MB	61	74.6	79%	Trekking; Samo	RP	CR	At surgeons discretion	2
			FB	58	73.9		Multigen Plus; Lima				
Tienboon 2012[77]	Thailand	Level II	MB	100	69.9	85.5%	PFC Sigma; DePuy	RP	NR	All resurfaced	2
			FB	100	68.4						
Wolterbeek 2012[87]	Netherlands	Level I	MB	9	63	65%	Triathlon; Stryker	RP	PS	None	1
			FB	11	66						
Kalisvaart 2012 [35]	USA	Level I	MB	76	67.4	70%	PFC Sigma; DePuy	RP	PS	All resurfaced	5
			FB	76	67.1						
Kim 2012 [37]	South Korea	Level II	MB	40	68	96.3%	PFC Sigma; DePuy	RP	PS	NR	2.5
			FB	40	66		NexGen LPS; Zimmer				
Shemshaki 2012[73]	Iran	Level I	MB	150	68	64%	PFC Sigma; DePuy	RP	PS	All resurfaced	5
			FB	150	70						
Jacobs 2011[33]	Netherlands	Level I	MB	46	67.6	70.7%	BalanSys; Mathys Medical	RP + AP gliding	CR	None	1
			FB	46	66.7						
Tibesku 2011[76]	Germany	Level II	MB	16	65	63.6%	Genesis II; Smith & Nephew	RP	CR	None	2
			FB	17	66						
Lampe 2011[47]	Germany	Level I	MB	48	70	73%	Columbus; B.Braun-Aesculap	RP	CR	None	1
			FB	52	69						
Woolson 2011[88]	USA	Level I	MB	33	78	NR	LCS; DePuy	RP	PS	All resurfaced	11.5
			FB	30	77.9		NexGen; Zimmer				
Ball 2011 [8]	USA	Level I	MB	51	64.9	56.0%	Scorpio; Stryker	RP	PS	NR	4
			FB	42	64						
Rahman 2010 [68]	Canada	Level I	MB	24	62.6	62.7%	PFC Sigma; DePuy	RP	PS	At surgeons discretion	3.5
			FB	27	62						
Munro 2010 [56]	New Zealand	Level I	MB	25	67.2	43.75%	PFC Sigma; DePuy	RP	NR	At surgeons discretion	2
			FB	23	67.7						
Hanusch 2010 [25]	UK	Level I	MB	50	70	49.5%	PFC Sigma; DePuy	RP	CR	None	1.1
			FB	55	69.4						
Matsuda 2010[52]	Japan	Level I	MB	30	73	77.0%	NexGen LPS; Zimmer	RP	PS	All resurfaced	5.7
			FB	31	76						
Gioe 2009[24]	USA	Level I	MB	176	71.8	2.8%	PFC Sigma; DePuy	RP	PS	All resurfaced	3.5
			FB	136	72.62						
Kim 2009[44]	South Korea	Level I	MB	92	69.5	92.4%	PFC Sigma; DePuy	RP	CR	All resurfaced	2.6
			FB	92			Advance medial pivot; Wright Medical				
Kim 2009[41]	South Korea	Level I	MB	61	48.3	73.8%	LCS; DePuy	MeBe	CR	All resurfaced	10.8
			FB	61			AMK; DePuy				
Vasdev 2009[83]	India	Level I	MB	60	63	58.3%	LCS; DePuy	RP	NR	None	3.5
			FB	60			NexGen LPS; Zimmer				
Wohlrab 2009[86]	Germany	Level II	MB	30	65.5	56.7%	NexGen; Zimmer	RP	**PS**	All resurfaced	5
			FB	30							
Harrington 2009[26]	USA	Level II	MB	68	63.7	64.3%	PFC Sigma; DePuy	RP	CR or PS	All resurfaced	2
			FB	72	63.3						
Hasegawa 2009[27]	Japan	Level I	MB	25	73	88%	PFC Sigma; DePuy	RP	PS	All resurfaced	3.3
			FB	25							
Higuchi 2009[30]	Japan	Level II	MB	31	68.4	72.1%	PFC Sigma; DePuy	RP	CR	NR	4
			FB	45							
Lädermann 2008[46]	Switzerland	Level I	MB	52	72	67.3%	PFC Sigma; DePuy	RP	PS	All resurfaced	7.1
			FB	52	69.8						
Wylde 2008[89]	UK	Level I	MB	118	68.9	54.5%	Kinemax Plus; Stryker	-	NR	At surgeons discretion	2
			FB	132	67.6						
Breugem 2008[11]	Netherlands	Level I	MB	48	71.2	64.1%	NexGen LPS; Zimmer	RP	PS	All resurfaced	1
			FB	55	68.9						
Kim 2007[45]	South Korea	Level I	MB	146	69.8	94.5%	LCS; DePuy	RP	PS	All resurfaced	13.2
			FB	146			AMK; DePuy		CR		
Kim 2007[40]	South Korea	Level I	MB	174	67	64.4%	PFC Sigma; DePuy	RP	CR	All resurfaced	5.6
			FB	174			PFC Sigma; DePuy				
Henricson 2006[29]	Sweden	Level I	MB	26	72	62.5%	MBK; Zimmer	RP + AP gliding	CR	At surgeons discretion	2
			FB	26			NexGen LPS; Zimmer				
Garling 2005[23]	Netherlands	Level II	MB	21	66	63.6%	Interax; Stryker	RP + AP gliding	PS	All resurfaced	2
			FB	21							
Aglietti 2005[2]	Italy	Level II	MB	103	71	83.8%	MBK; Zimmer	RP + AP gliding	CR	All resurfaced	3
			FB	107	69.5		NexGen LPS; Zimmer		PS		
Bhan 2005[9]	India	Level I	MB	16	63	68.8%	LCS; DePuy	RP	**PS**	None	6
			FB	16			Columbus; Zimmer				
Pagnano 2004[61]	USA	Level II	MB	80	67	69.6%	PFC Sigma; DePuy	RP	**PS**	All resurfaced	1
			FB	160							
Saari 2003[69]	Sweden	Level II	MB	7	69	81%	Freeman-Samuelson, Finsbury	RP	CR or PS	NR	1
			FB	15							
Price 2003[66]	UK	Level I	MB	21	73.1	60%	TMK; Biomet	RP + AP gliding	**CR**	None	1
			FB	19			ACG; Biomet				
Kim 2001[42]	South Korea	Level I	MB	120	65	69%	LCS; DePuy	MeBe	**PS**	All resurfaced	7.4
			FB	120			AMK; DePuy				

*LoE* Level of evidence; *TKA* total knee arthroplasty; *MB* mobile-bearing; *FB* fixed-bearing; *RP* rotating platform; *RP + AP* rotating platform and anterior–posterior gliding; *MeBe* meniscal bearing; *CR* cruciate-retaining; *PS* posterior-stabilized; *NR* not-reported

### Quality assessment

Low risk of bias was found in 27 studies, some concern for bias in 28 studies and high risk of bias in the remaining 15 studies. Most studies had a low risk of bias for deviation from intended interventions, missing outcome data, measurement of outcomes and in the selection of reported results. In terms of randomization, 55.7% of included studies had a low risk of bias, 38.5% had some concern for bias, and 5.7% had a high risk for bias. A graphic summary of the qualitative assessment is displayed in Supplementary Fig. 1.

### Revision Rates

**Fig. 2:**
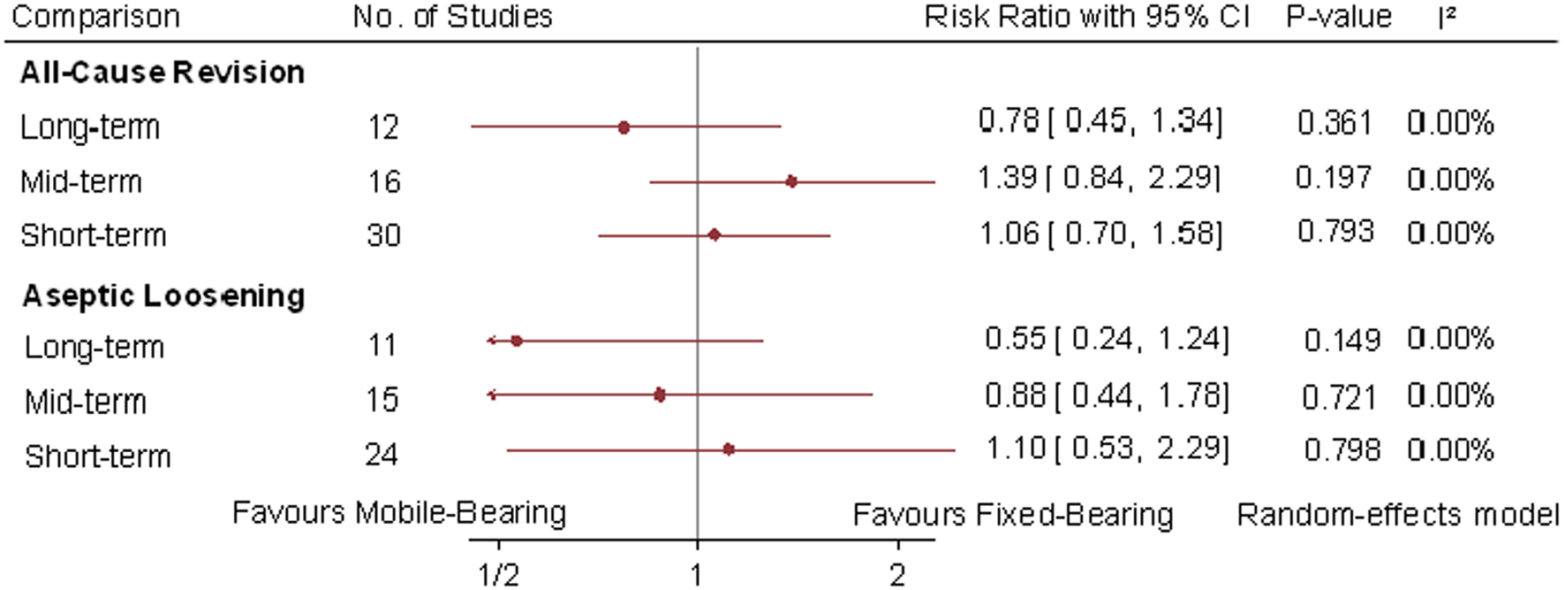
Random-effect meta-analytic comparison for all-cause revision and aseptic loosening between mobile-bearing versus fixed-bearing total knee arthroplasty. CI: confidence interval

Revisions were reported in 58 studies, with 2.4% (96 out of 3978) revision rates in mobile-bearing TKA and 2.2% (88 out of 3947) revision rate in fixed-bearing TKA. The all-cause revision rates were not statistically significant when comparing mobile-bearing versus fixed-bearing TKA at short-term (RR 1.06; 95% CI 0.7, 1.58; P = 0.793; I^2^ = 0%), mid-term (RR 1.39; 95% CI 0.84, 2.29; P = 0.197; I^2^ = 0%) and long-term (RR 0.78; 95% CI 0.45, 1.34; P = 0.361; I^2^ = 0%) follow-up intervals. Likewise, among 5 studies there was no significant difference in aseptic loosening at the three follow-up intervals (Fig. 2).

### Functional Scores

**Fig. 3:**
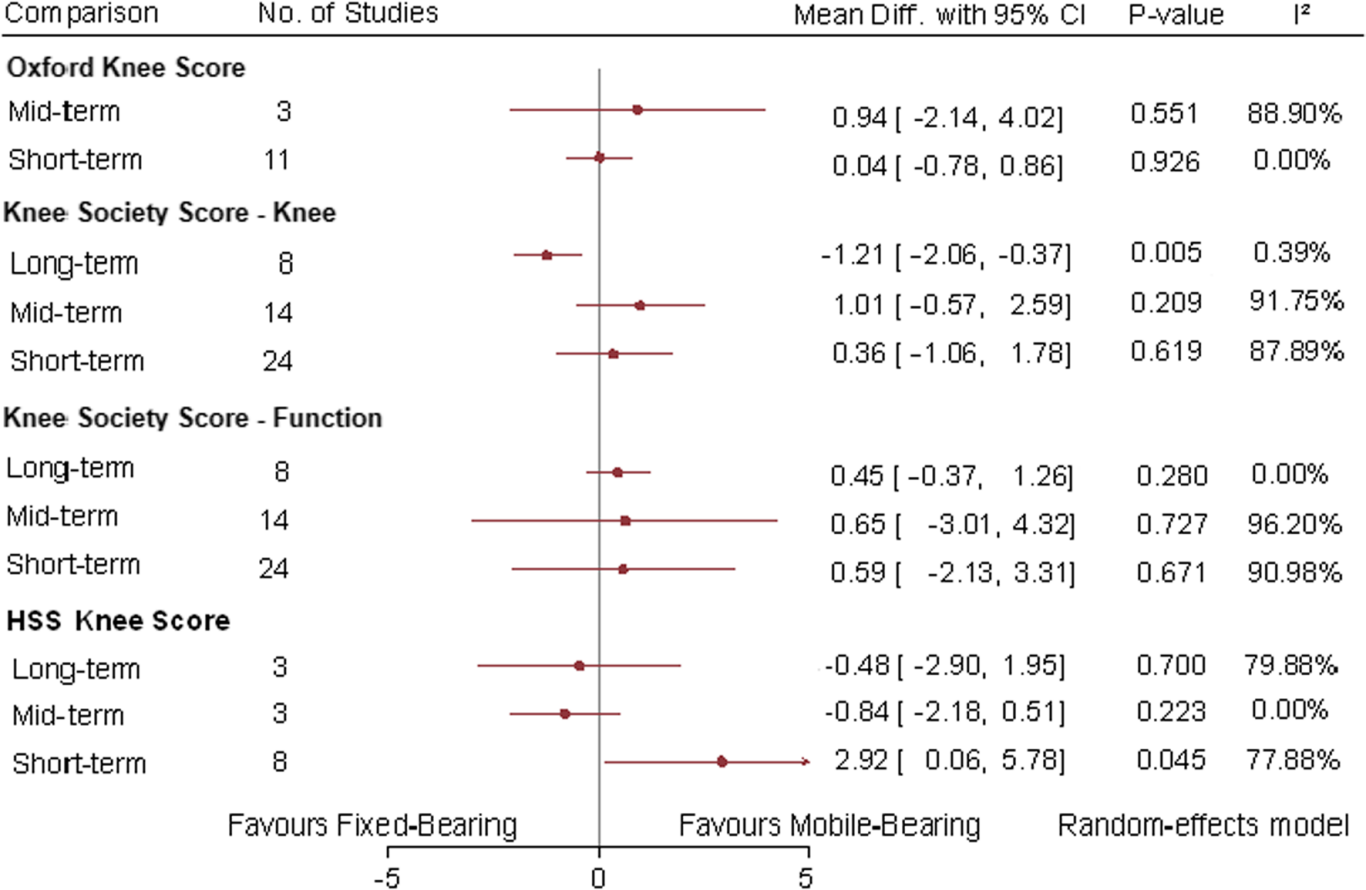
Random-effect meta-analytic comparison for functional knee scores between mobile-bearing versus fixed-bearing total knee arthroplasty. CI: confidence interval

Eleven and 3 studies reported the OKS at short and mid-terms, respectively. There was no significant difference between mobile-bearing and fixed-bearing TKA at both short term (MD 0.04; 95% CI −0.78, 0.86; P = 0.926; I^2^ = 0%) and mid-term (MD 0.94; (95% CI −2.14, 4.02; P = 0.551; I^2^ = 88.9%) (Fig. 3).

The KSS knee and function sub-scores were reported in 24 studies at short-term, 14 studies at mid-term and 8 studies at long-term follow-up. There was no statistically significant difference between mobile-bearing and fixed-bearing TKA at short term (MD 0.36; 95% CI −1.06, 1.78; P = 0.619; I^2^ = 87.89%) and mid-term (MD 1.00; 95% CI −0.57, 2.59; P = 0.209; I^2^ = 91.75%) for the KSS knee sub-score. The long-term follow-up demonstrated statistically significant better KSS knee sub-score in favor of fixed-bearing TKA (MD −1.21; 95% CI −2.06, −0.37; P = 0.005; I^2^ = 0.39%). Regarding the functional KSS sub-score, there were no statistically significant differences at short-term (MD 0.59; 95% CI −2.13, 3.31; P = 0.671; I^2^ = 90.98%), mid-term (MD 0.65; 95% CI −3.01, 4.32; P = 0.727; I^2^ = 96.2%) and long-term (MD 0.45; 95% CI −0.37, 1.26; P = 0.28; I^2^ = 0%) follow-ups between mobile-bearing and fixed-bearing TKA. Figure 3 displays the KSS sub-score comparisons.

The HSS knee score was reported in 8 studies at short term, 3 studies at mid-term and 3 studies at long term. The short-term follow-up comparison demonstrated slightly better HSS scores in favor of mobile-bearing TKA (MD 2.92; 95% CI 0.06, 5.78; P = 0.045; I^2^ = 77.88%). The mid-term (MD −0.84; 95% CI −2.18, 0.51; P = 0.223; I^2^ = 0%) and long-term (MD −0.48; 95% CI −2.9, 1.95; P = 0.7; I^2^ = 79.88%) follow-up intervals did not demonstrate any statistically significant difference for the HSS knee scores (Fig. 3).

**Fig. 4:**
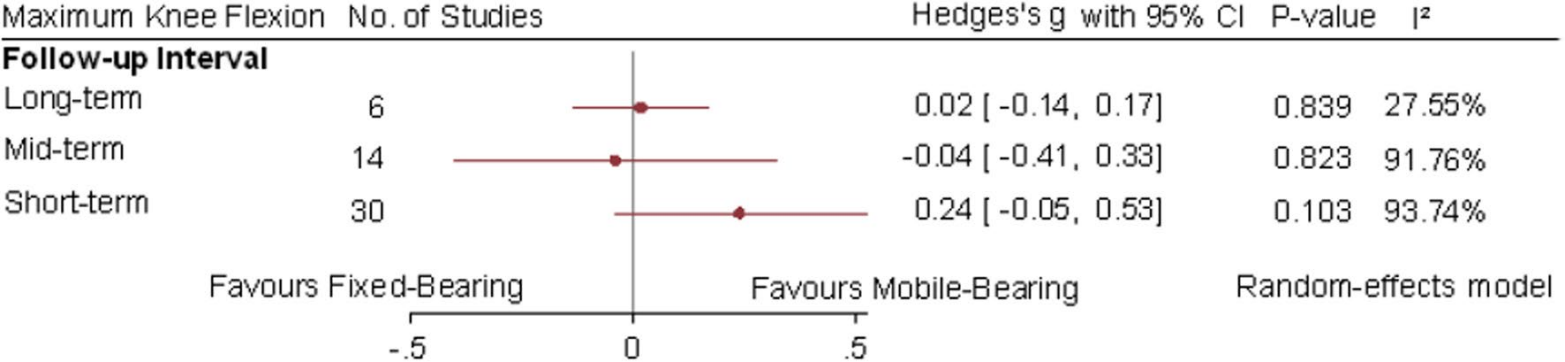
Random-effect meta-analytic comparison for maximum knee flexion between mobile-bearing versus fixed-bearing total knee arthroplasty. CI: confidence interval

The range of motion was reported in 27 studies at short term, 12 studies at mid-term and 6 studies at long term. No differences were significant between mobile-bearing and fixed-bearing TKA at any of the three follow-up intervals (Fig. 4).

### Radiographic outcomes

**Fig. 5:**
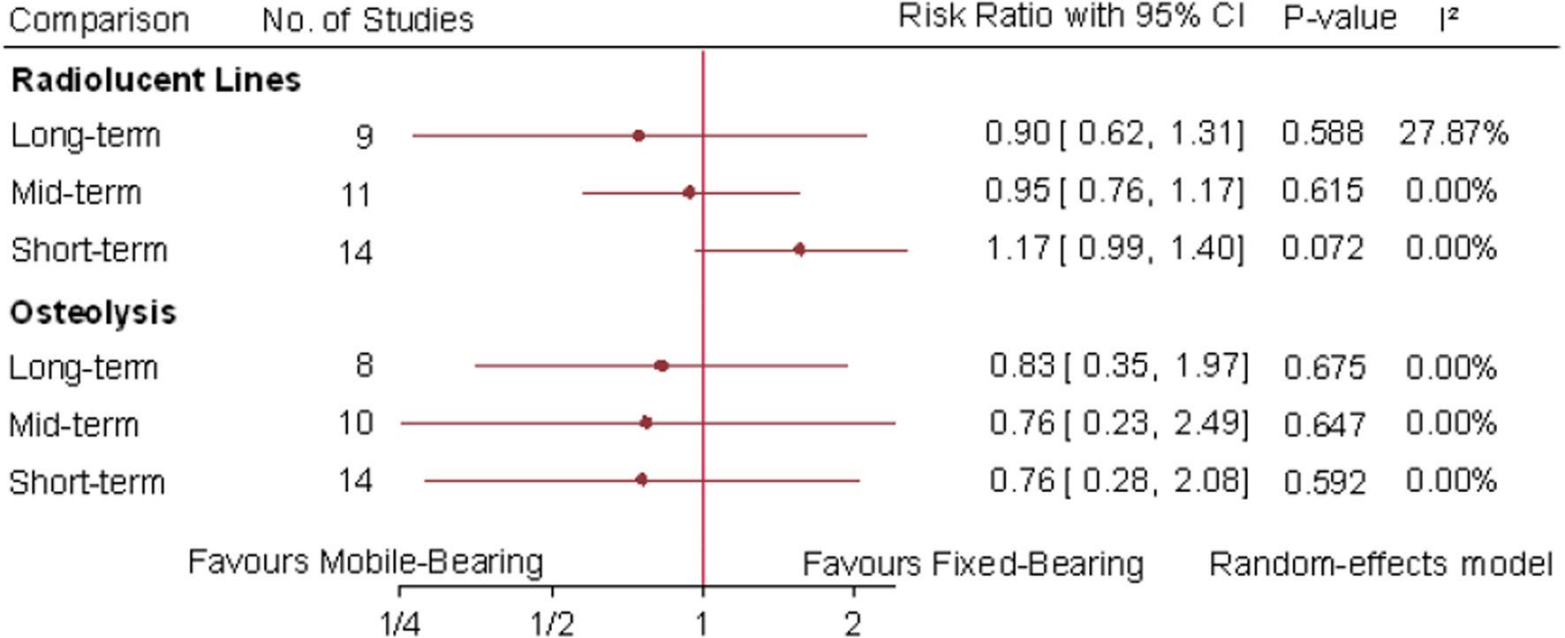
Random-effects meta-analytic comparison for radiolucent lines and osteolysis between mobile-bearing versus fixed-bearing total knee arthroplasty. CI: confidence interval

Radiolucent lines were pooled in 14 studies at short-term, 11 studies at mid-term and 9 studies at long-term follow-up intervals. There was no statistically significant difference at short-term (RR 1.17; 95% CI 0.99, 1.4; P = 0.072; I^2^ = 0%), mid-term (RR 0.95; 95% CI 0.76, 1.17; P = 0.615; I^2^ = 0%) or long-term (RR 0.9; 95% CI 0.62, 1.31; P = 0.588; I^2^ = 27.87%) intervals between mobile-bearing and fixed-bearing TKA (Fig. 5).

Osteolysis was pooled in 14 studies at short-term, 10 studies at mid-term and 8 studies at long-term follow-up intervals. Meta-analytic comparison of mobile-bearing TKA with fixed-bearing TKA failed to demonstrate any statistically significant difference at short-term (RR 0.76; 95% CI 0.28, 2.08; P = 0.592; I^2^ = 0%), mid-term (RR 0.768; 95% CI 0.23, 2.49; P = 0.647; I^2^ = 0%) and long-term intervals (RR 0.83; 95% CI 0.35, 1.97; P = 0.675; I^2^ = 0%) (Fig. 5).

## Discussion

This meta-analysis on randomized controlled trials demonstrated no significant difference between mobile-bearing and fixed-bearing TKA with regard to all outcome measures compared. The revision rates among studies throughout all follow-up intervals were 2.4% in mobile-bearing TKA and 2.2% in fixed-bearing TKA. Furthermore, this meta-analysis did not result in statistically significant differences in revision rates or aseptic loosening between both designs at short-term, mid-term and long-term follow-up intervals. The long-term follow-up interval ranged from 10 to 17 years postoperatively in 12 studies for revision rates and 11 studies for aseptic loosening. Likewise, previous meta-analyses and the vast majority of included randomized trials found similar survivorship when comparing mobile-bearing and fixed-bearing TKA [55, 81]. In contrast, few non-randomized studies have found contradicting evidence. A registry-based prospective study by Namba et al. [57] on 47,339 knees found that mobile-bearing TKA had a twofold increase in aseptic revision at 6.7 years when compared to fixed-bearing TKA following a multi-variate adjusted regression analysis (P < 0.001). Likewise, Heesterbeek et al. [28] found in a recent multicenter retrospective study that fixed-bearing had superior survivorship at 12 years as opposed to mobile-bearing designs. In a randomized trial by Fransen et al. [21], mobile-bearing TKA was found to have a 6-times higher risk for all-cause revision compared to fixed-bearing TKA at 5-year follow-up. This study had major limitations such as a 38% drop-out rate and lack of blinding of those who assessed outcomes.

Assessment of knee functional outcomes demonstrated no clinically significant differences between mobile-bearing and fixed-bearing TKAs. The OKS was only pooled at the short- and the mid-term follow-up intervals without any statistical significance. The KSS knee sub-score was not statistically significant at the short- and the mid-term follow-up intervals; however, at the long-term there was a statistically significant effect in favor of fixed-bearing TKA. It is paramount to acknowledge that this finding was not clinically significant as the minimal clinically important difference (MCID) of the KSS knee sub-score is between 5.3 and 5.9 points [48]. The KSS functional sub-score was statistically insignificant at short-, mid- and long-term follow-ups. The HSS knee score was in favor of mobile-bearing TKA at the short-term follow-up which was statistically significant, however, yet clinically irrelevant as the HSS MCID is 8.29 points [32]. The mid- and the long-term follow-up for the HSS knee score had no statistically significant difference between mobile-bearing and fixed-bearing TKA. Furthermore, there was no statistically significant difference between mobile-bearing and fixed-bearing TKA for the postoperative maximum knee flexion. Most prior meta-analyses and randomized trials have shown similar results without any statistical difference in clinical outcomes. Nonetheless, several studies have had better outcomes with mobile-bearing TKA. At 6–10-year follow-up, the randomized trial Baktir et al. [7] resulted in significantly improved pain and KSS knee sub-scores in mobile-bearing TKA. However, the authors found no difference in the functional sub-score of the KSS. In a recent randomized trial by Powell et al. [64], mobile-bearing TKA had superior results with the OKS and the Knee Injury and Osteoarthritis Outcome Score sports and quality of life subscales. This difference was observed at 10-year follow-up which exceeded the MCID threshold. In contrast, a similarly well-designed trial by Abdel et al. [1] refuted such findings without any advantages provided by the mobile-bearing design over fixed-bearing TKA in terms of maximum knee flexion or function at 10-year follow-up.

In terms of radiological outcomes, no significant differences were detected between both mobile-bearing and fixed-bearing TKA at the short-, mid- and long-term follow-up intervals for either radiolucent lines or osteolysis. In all randomized trials included except for the study by Bailey et al. [6], there was no statistical difference between mobile-bearing and fixed-bearing designs in radiological outcomes. Bailey et al. [6] have reported that radiolucency was higher in the mobile-bearing designs around the tibial component; however, this was clinically insignificant. Furthermore, in a radiostereometric analysis (RSA) by Schotanus et al. [71] both mobile-bearing and fixed-bearing designs had similar implant migration detected by the maximum total point motion at 2 years.

The strengths of this study were the inclusion of the largest number of randomized trials thus far, and the analyzing outcomes measure at the short-, mid- and long-term follow-up intervals. To the best of our knowledge, this is the most comprehensive recent meta-analysis on the topic. The last systematic review was performed in 2017 by Fransen et al. [22]. In addition, the last two meta-analyses were performed in June 2020 on this topic by Chen et al. [15] and Wang et al. [84]; however, both meta-analyses combined had 16 randomized trials versus 70 randomized trials in our meta-analysis. Furthermore, both meta-analyses had conflicting results as one supported long-term outcomes of mobile-bearing TKA, yet the other found no difference between fixed-bearing and mobile-bearing designs. In contrast, our study found no differences between mobile- and fixed-bearing designs at anytime point; this is mainly due to pooling data from 70 RCTs, thereby demonstrating more valid results. Several limitations to this meta-analysis should be acknowledged. Although we included RCTs, several trials had high risk of bias as evident in our qualitative review. Another limitation was that outcome measures varied among included studies, which prevented measuring the long-term outcome using the OKS and pooling a higher number of patients in other outcome measures. Implant migration using RSA was not analyzed due to the variability in its reporting across RSA-based studies. Another important limitation was that different types of mobile-bearing TKA were used by different trials, in turn this could be a potential source of bias given the mobile-bearing type was not adjusted for.

## Conclusion

This meta-analysis on 70 randomized controlled trials demonstrated no clinically significant differences between mobile-bearing and fixed-bearing TKA at short-, mid- and long-term follow-up for revision rates, aseptic loosening rates, knee functional scores, maximum knee flexion and radiographic lucent lines and osteolysis. The current level of evidence demonstrated that both mobile-bearing and fixed-bearing designs achieved excellent outcomes, yet it does not prove the theoretical advantages of the mobile-bearing insert over its fixed-bearing counterpart. Given that the use of either design can be supported by this meta-analysis, we recommend that surgeons can use mobile- or fixed-bearing inserts in TKA at their own discretion.

## Supplementary information

Below is the link to the electronic supplementary material.

Supplementary file1 (DOCX 36.0 kb)

## Data Availability

Not applicable.
