# Supplementary figures and images for: Mobile-bearing versus fixed-bearing total knee arthroplasty: a meta-analysis of randomized controlled trials
Source: Eur J Orthop Surg Traumatol. 2021 May 22;32(3):481–95. doi: 10.1007/s00590-021-02999-x (PMC8924090; doi:10.1007/s00590-021-02999-x)

Supplementary Figure 1: Qualitative Analysis of Included Studies.


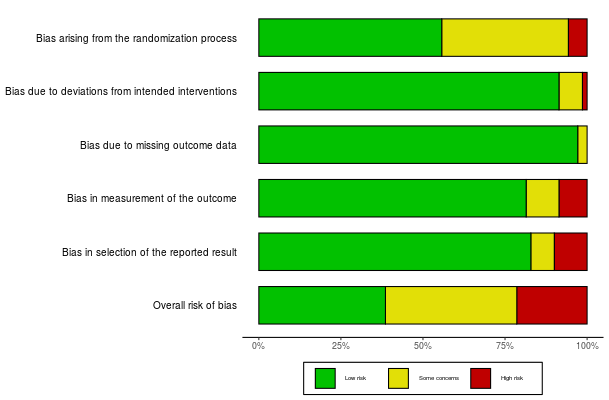

Supplement: Supplementary file 1 — Supplementary file1 (DOCX 36.0 kb) [file 590_2021_2999_MOESM1_ESM.docx]
